# Supplementary material for: Context dependent effects of ascorbic acid treatment in TET2 mutant myeloid neoplasia
Source: Commun Biol. 2020 Sep 7;3:493. doi: 10.1038/s42003-020-01220-9 (PMC7477582; doi:10.1038/s42003-020-01220-9)
Supplement: Supplementary file 1 — Description of Additional Supplementary Files [file 42003_2020_1220_MOESM1_ESM.pdf]

Descriptions of Additional Supplementary Files

Supplementary Data 1. Source Data for Figure 1 - Figure 6 and Supplementary Figure 1 - Supplementary Figure 3 and Supplementary Figure 5.

Supplementary Data 2. Characteristics of primary cells and cell lines

Supplementary Data 3. TET2 mutant MN patient informations

Supplementary Data 4. TET2 lysine missense mutation in 4930 patients with myeloid neoplasia

Supplementary Data 5. Detailed list of primers and their sequences
