# Supplementary material for: Context dependent effects of ascorbic acid treatment in TET2 mutant myeloid neoplasia
Source: Commun Biol. 2020 Sep 7;3:493. doi: 10.1038/s42003-020-01220-9 (PMC7477582; doi:10.1038/s42003-020-01220-9)
Supplement: Supplementary file 2 — Supplementary Information [file 42003_2020_1220_MOESM2_ESM.pdf]

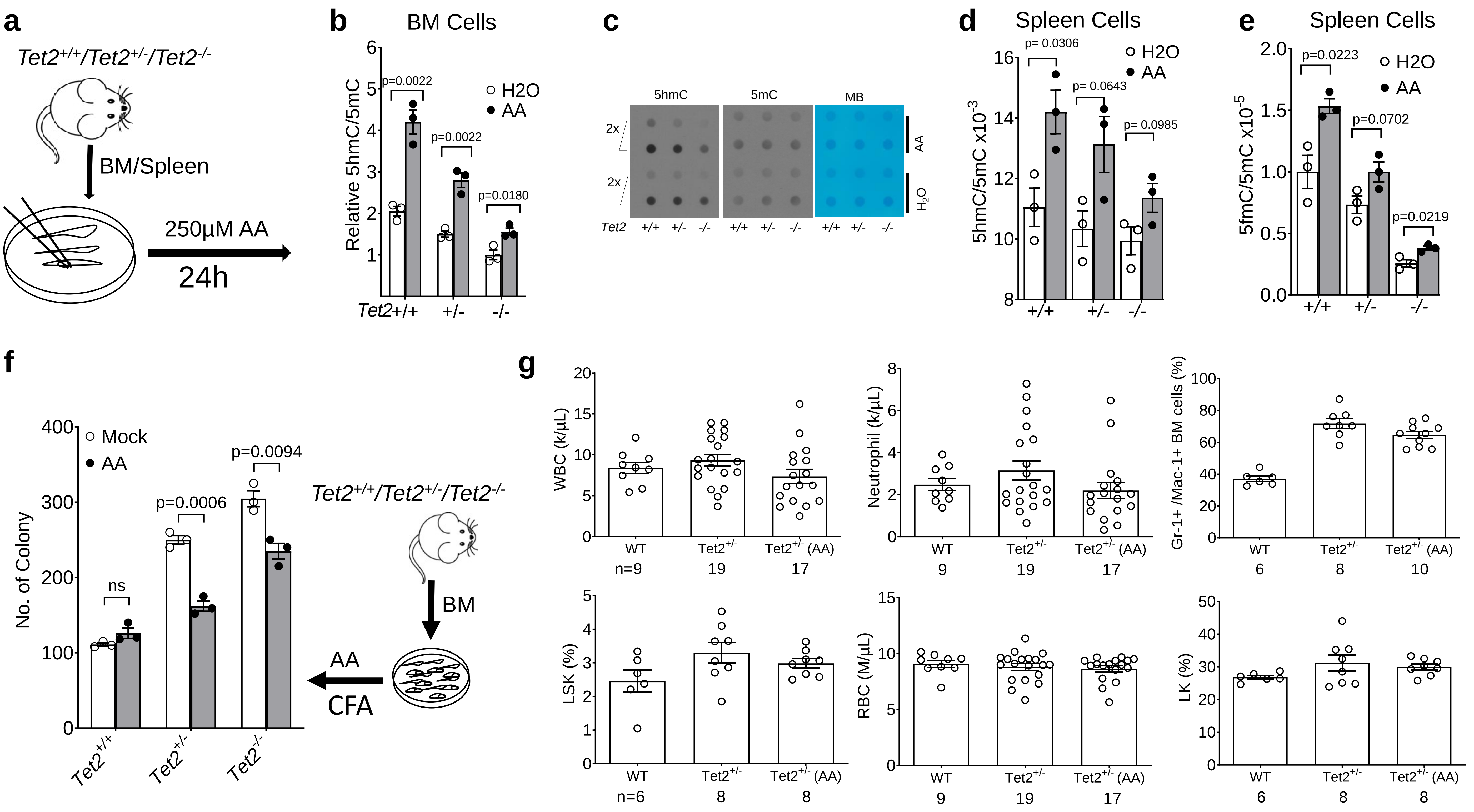

Supplementary Figure 1. Ascorbic acid increases TET-dependent oxidation products (TDOP) in cells. a-e. Isolated mononuclear cells from *Tet2*$^{+/+}$, *Tet2*$^{+/-}$, and *Tet2*$^{-/-}$ mice BM (**b-c**) and spleen (**d-e**) were treated at concentration of 1 million per ml with 250µM AA or H_2O. Genomic DNA was extracted after 24 hours of treatment. DNA oxidation products were assessed by dot blot (**b-c**) or 2D-UPLC-MS/MS (**d-e**). F. *Tet2*$^{+/+}$, *Tet2*$^{+/-}$, and *Tet2*$^{-/-}$ mouse BM cells treated for 24h with 250 µM AA or H_2O and proliferation was assessed after 14 days by colony forming assay. **b**, **d**, **e** and **f**. Data are shown as mean with SEM of triplicate and are representative of at least two independent experiments; statistical significance (p-values) from two tailed t-test are indicated; ns: not significant. **g**. *Tet2*$^{+/-}$ mice treated with or without AA for one year were sacrificed for the following analyses: WBC, Neutrophils, Gr-1$^+$ and/or Mac-1$^+$ cells, LSK cells, RBCs and LK cells. Data are shown with mean ± SEM. There is no statistically significant difference between the means of *Tet2*$^{+/-}$ and *Tet2*$^{+/-}$+AA.

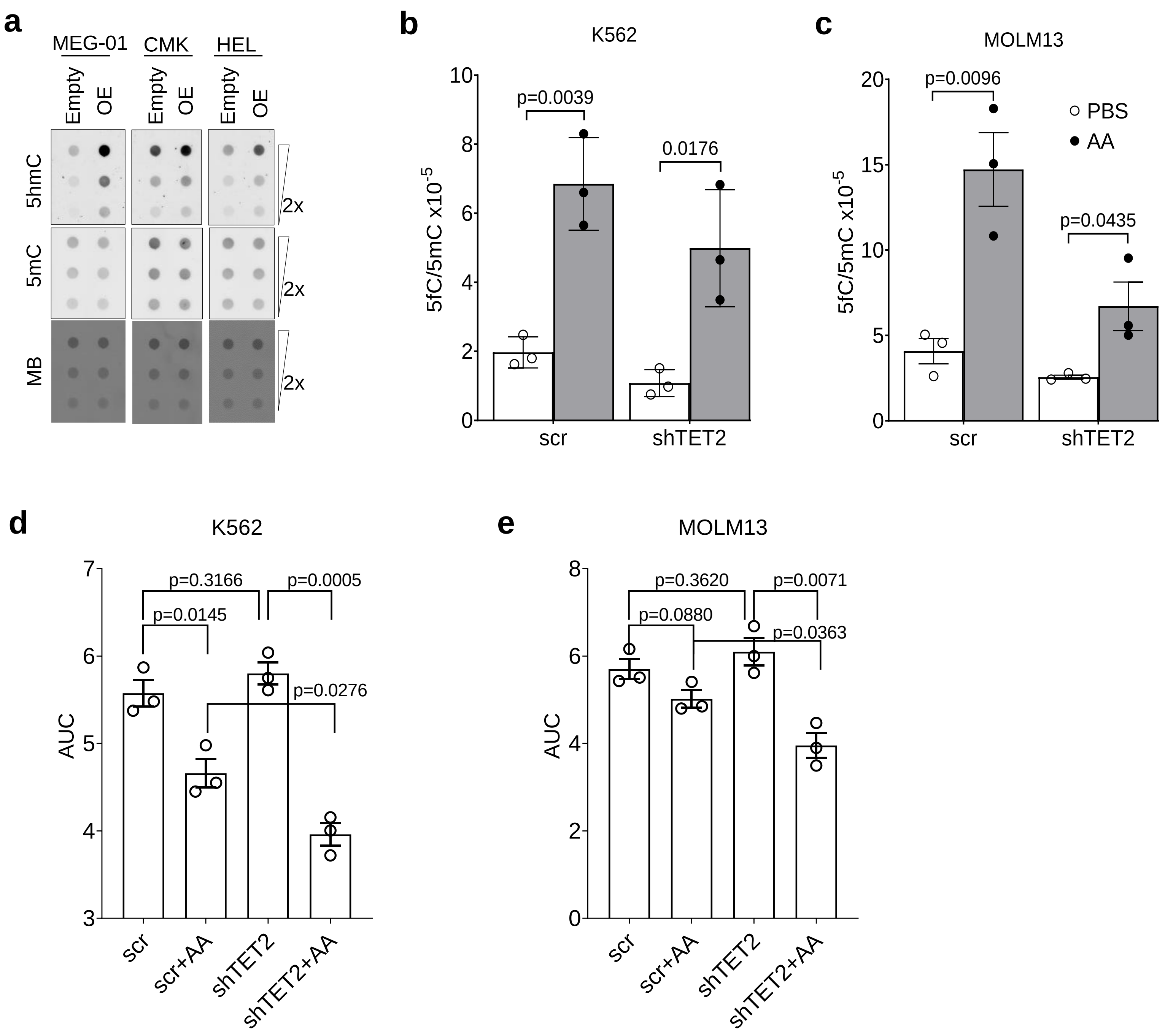

Supplementary Figure 2. TET2 activity correlates with DNA oxidation levels. a. MEG-01, CMK, and HEL cell lines were stably transfected with either an empty pOZ vector or a pOZ-TET2-overexpression ($TET2^{OE}$) vector. 5hmC and 5mC levels were assessed by dot blot assay. **b-c**. Human K562 and MOLM-13 cells were treated with scrambled shRNA (scr, $TET2^{WT}$) or shRNA targeting TET2 ($TET2^{KD}$). $TET2^{WT}$ and $TET2^{KD}$ cells were treated for 24 hours with either H_2O or 250 µM AA; DNA oxidation products were assessed by 2D-UPLC-MS/MS. **d-e**. Area under the curve (AUC) was calculated and analyzed for each curve in **Figs. 3g-h** in GraphPad Prism 8. Data are shown as mean ± SEM of triplicate and are representative of two independent experiments; statistical significance (p-values) from two tailed t-test are indicated.

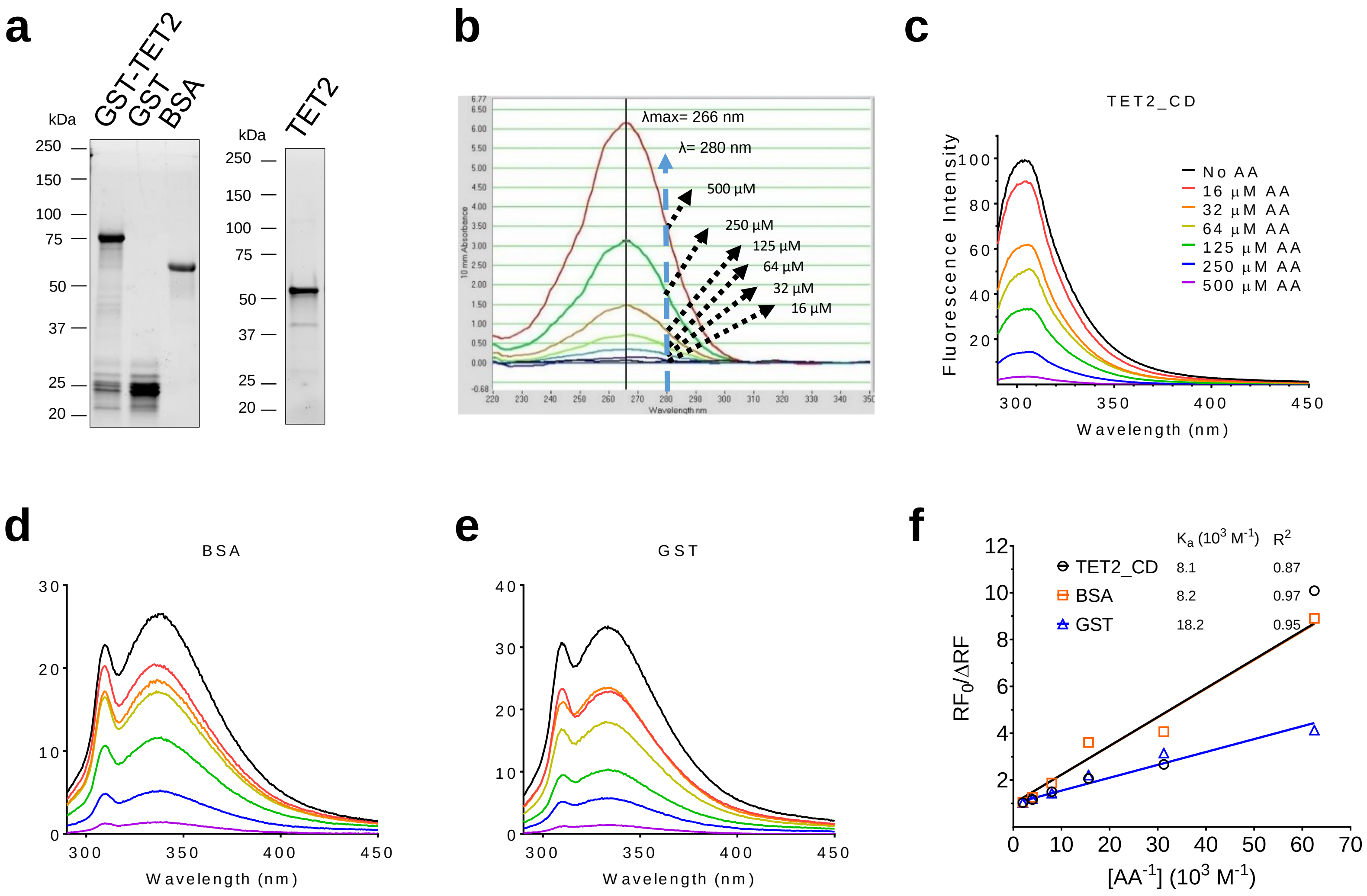

Supplementary Figure 3. UV-induced fluorescence quenching based binding for AA to disparate proteins. **a**. Gel staining (TGX Stain-Free™ Precast Gels, BIO-RAD) of purified proteins. **b**. AA was dissolved in water and absorbance was measured by NanoDrop 1000 Spectrophotometer, Thermo Scientific. **c**-**e**. Fluorescence quenching of proteins by AA at different concentrations. **f**. Modified Stern-Volmer plot for estimating the binding constant of AA and proteins. RF_0 and RF are fluorescence intensities of proteins in the absence and presence of AA, respectively. ΔRF equals RF_0-RF.

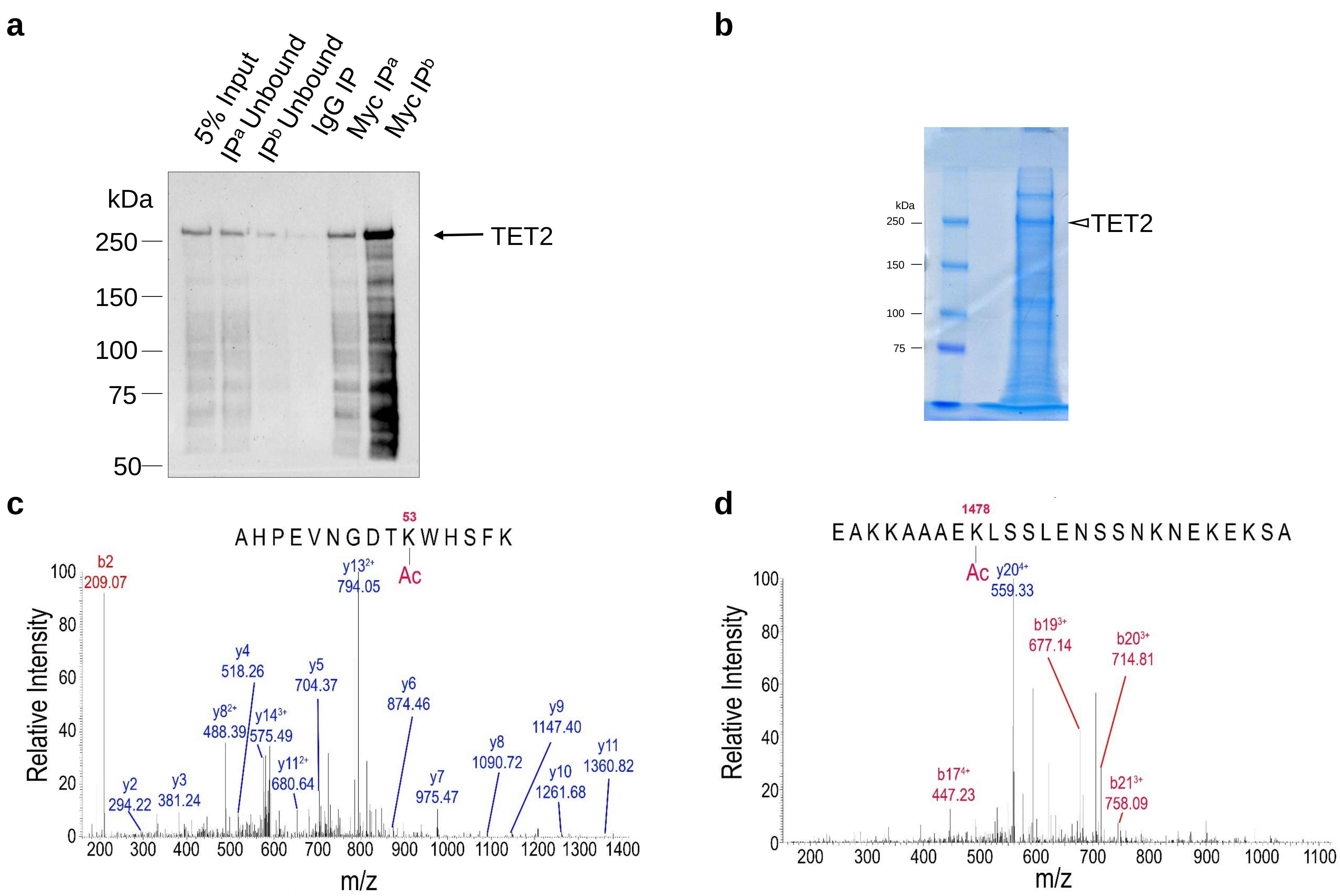

Supplementary Figure 4. a. Western blot analysis of TET2 immunoprecipitation (IP) by anti Myc tag antibody. Myc-TET2 was ectopically expressed in HEK293T cells by plasmid transfection. Protein was extracted for IP after 3 days. IgG IP: rabbit IgG + Protein G Magnetic Beads (Cell Signaling, 70024); Myc IPa: Myc-Tag antibody (Cell Signaling, 2278) + Protein G Magnetic Beads; Myc IPb: Myc-Tag antibody conjugated magnetic beads (Cell Signaling, 5698); Unbound: protein solution supernatant after binding with Myc Antibody + Magnetic Beads. **b**. Coomassie blue staining of immunoprecipitated TET2 after sodium dodecyl sulphate-polyacrylamide gel electrophoresis (SDS-PAGE). TET2 protein is indicated by arrow head. **c-d**. Mass Spectrometric analysis was performed for purified TET2 protein. K53 and K1478 were identified as acetylated lysine.

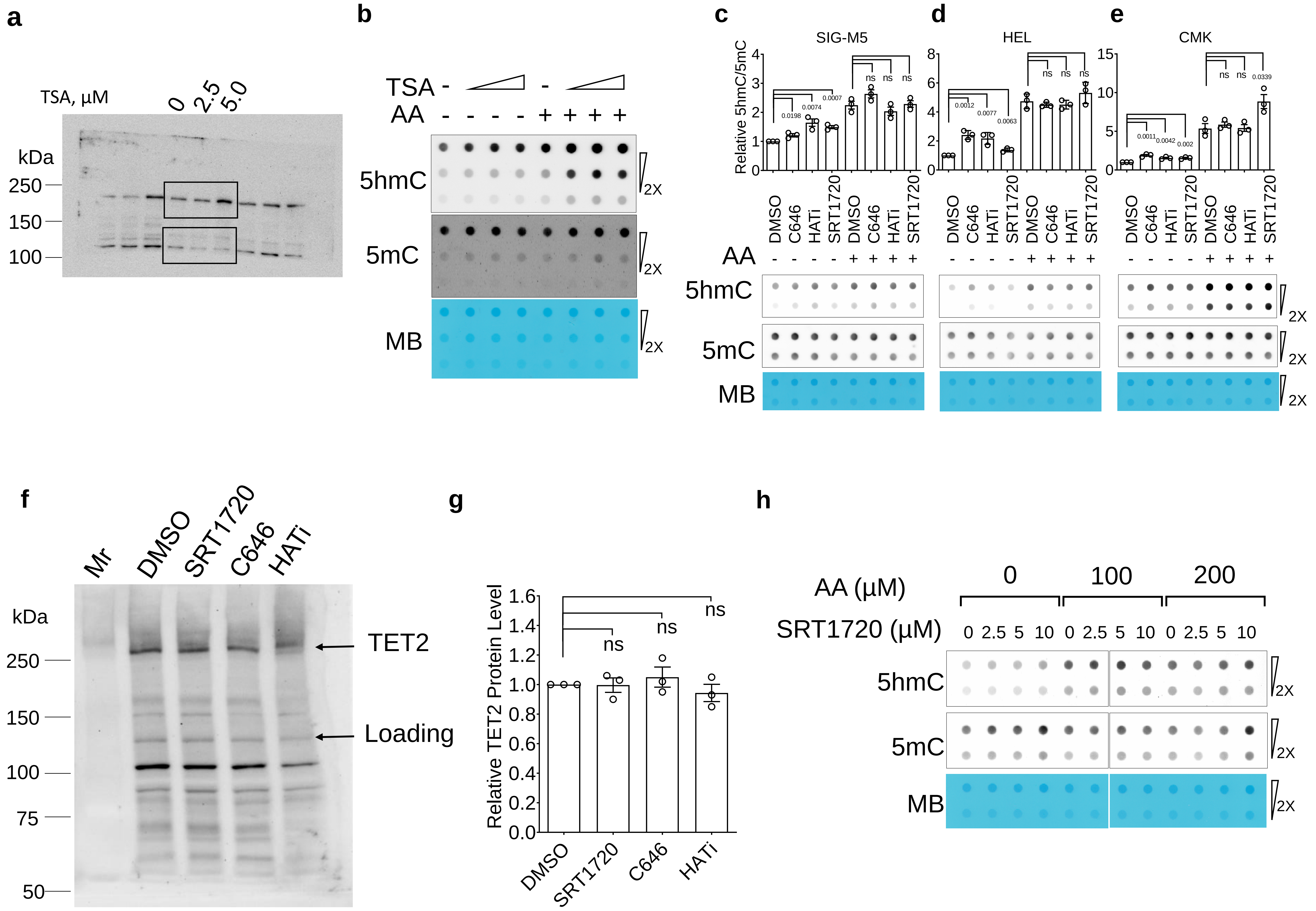

Supplementary Figure 5 Continued

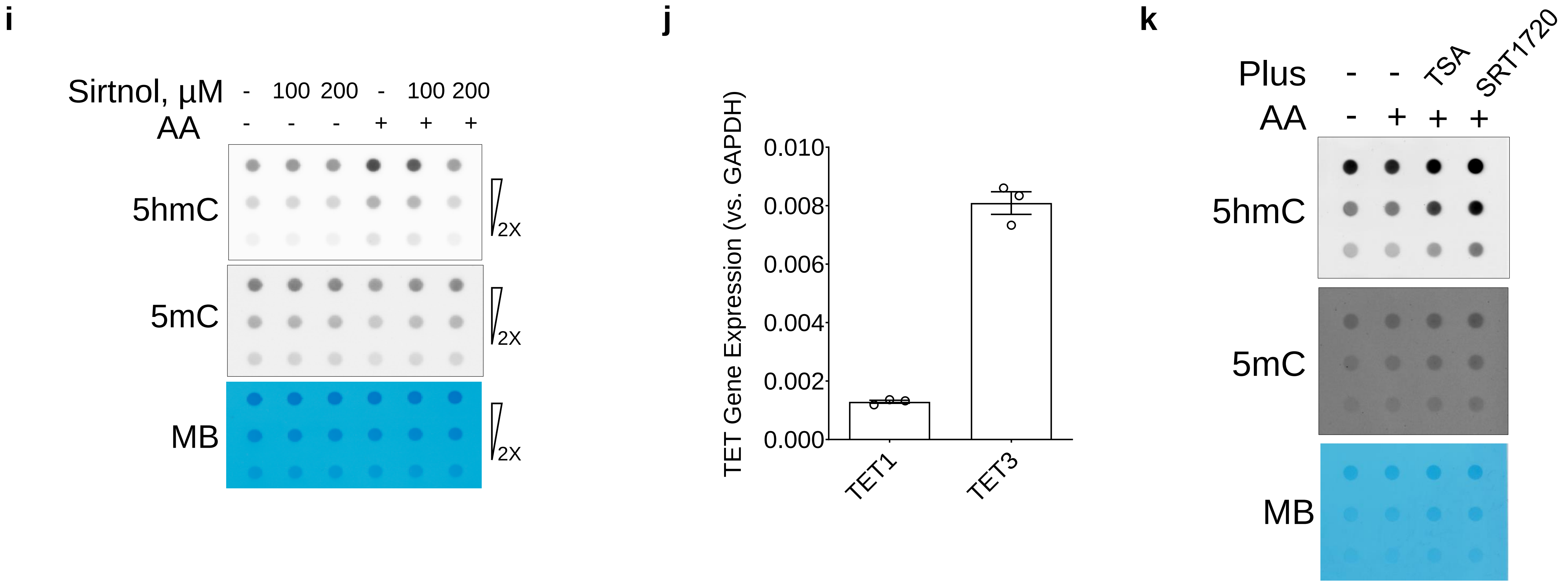

Supplementary Figure 5. Class I, II HDAC inhibitors or class III HDAC activators amplifies AA mediated TET-activity in cells compared. **a**. Original blot for figure 5A. **b**.CMK cells were treated for 12 hours with 0, 1.25, 2.5 or 5 µM of TSA (Trichostatin A, Cayman, Item No. 89730) in the presence or absence of 100 µM AA. Total genomic DNA was harvested and 5hmC/5mC were determined by dot blot analysis. **c**-**e**. SIG-M5, HEL and CMK cell lines were treated for 24 hours with DMSO, 20 µM C646 (Histone Acetyltransferase inhibitor, Selleckchem, Catalog #S7152), 20 µM HATi (Histone Acetyltransferase Inhibitor II, Cayman, Catalogue #19835), or 5 µM SRT1720 (Sirtuins activator, Cayman, Catalogue #10011020) in the presence or absence of 100 µM AA. 5hmC/5mC levels were assessed by dot blot. **f**-**g**. Western blot analysis and quantification of TET2 protein level after treatment of 20 µM C646, 20 µM HATi or 5 µM SRT1720 for CMK cells. Nuclear protein fractions were extracted for western blot. **h**. TET1/3 RNA quantification by qRT-PCR for SIG-M5 cell line. **i**. CMK cells were treated for 12 hours with 0-10 µM of SRT1720 and 0-200 µM AA. 5hmC/5mC levels were assessed by dot blot. **j**. Sirtinol, a known sirtuin inhibitor, prevents AA mediated TET activity. CMK cells were treated with increasing concentrations (0, 100 and 200 µM) of sirtinol in the presence and absence of 100 µM AA for 12 hours and 5hmC was determined by dot blot analysis. Cells were treated at the density of 5×10^5 cells/ml. **k**. $TET2^{+/-}$ mononuclear cells isolated from MN patient BMs were treated with 100 µM of AA or combined with either 5 µM of TSA or 5 µM of SRT1720. DNAs were extracted 24 hours after treatment for dot blot analyisis for 5hmC and 5mC. **c**-**e** and **g**-**h**. Data are shown as mean ± SEM of triplicate. Data are representative of two independent experiments; statistical significance (p-values) from two tailed t-test are indicated; ns: not significant. MB: methylene blue staining for loading control.

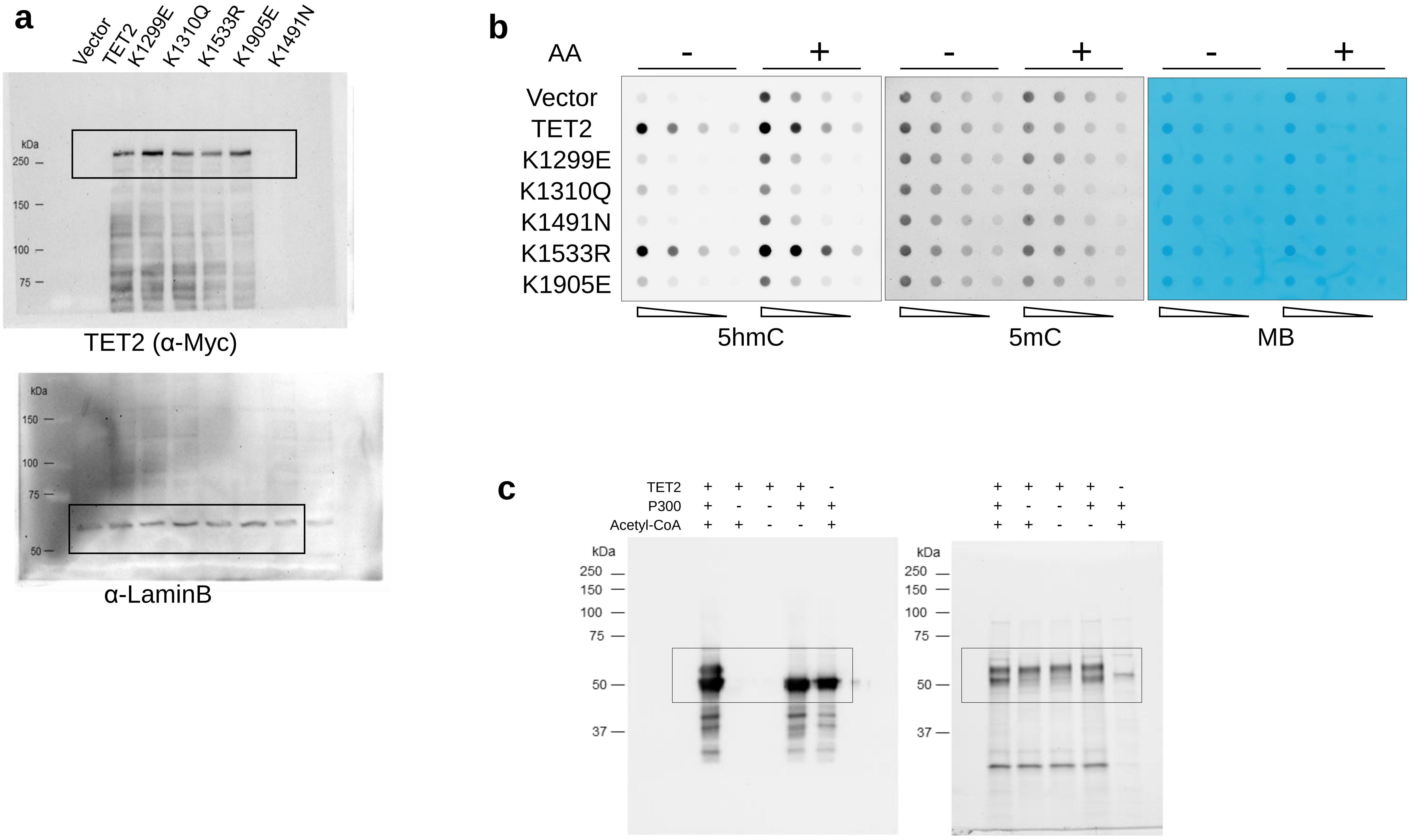

Supplementary Figure 6. a. Original blot for the data used in **Figure 6b**. **b**. Dot blot characterization of TET2 lysine mutations activity. Two days after ectopically expression of TET2 mutations, HEK293T cells were treated with 100 µM AA or left untreated. DNAs were extracted from harvested cells 24 hours after AA treatment. Data are representative of three independent experiments. **c**. Original blot for the data used in **Figure 6e**.
