# Supplementary material for: Context dependent effects of ascorbic acid treatment in TET2 mutant myeloid neoplasia
Source: Commun Biol. 2020 Sep 7;3:493. doi: 10.1038/s42003-020-01220-9 (PMC7477582; doi:10.1038/s42003-020-01220-9)
Supplement: Supplementary file 8 — Reporting Summary [file 42003_2020_1220_MOESM8_ESM.pdf]

natureresearch

Corresponding author(s): Jaroslaw Maciejewski, MD, PhD & Babal K jha, PhD

Last updated by author(s): Jul 23, 2020

Reporting Summary

Nature Research wishes to improve the reproducibility of the work that we publish. This form provides structure for consistency and transparency in reporting. For further information on Nature Research policies, see Authors & Referees and the Editorial Policy Checklist.

Statistics

For all statistical analyses, confirm that the following items are present in the figure legend, table legend, main text, or Methods section.

n/a	Confirmed	
☐	☒	The exact sample size (*n*) for each experimental group/condition, given as a discrete number and unit of measurement
☐	☒	A statement on whether measurements were taken from distinct samples or whether the same sample was measured repeatedly
☐	☒	The statistical test(s) used AND whether they are one- or two-sided<br>*Only common tests should be described solely by name; describe more complex techniques in the Methods section.*
☒	☐	A description of all covariates tested
☒	☐	A description of any assumptions or corrections, such as tests of normality and adjustment for multiple comparisons
☒	☐	A full description of the statistical parameters including central tendency (e.g. means) or other basic estimates (e.g. regression coefficient) AND variation (e.g. standard deviation) or associated estimates of uncertainty (e.g. confidence intervals)
☐	☒	For null hypothesis testing, the test statistic (e.g. *F*, *t*, *r*) with confidence intervals, effect sizes, degrees of freedom and *P* value noted<br>*Give P values as exact values whenever suitable.*
☒	☐	For Bayesian analysis, information on the choice of priors and Markov chain Monte Carlo settings
☒	☐	For hierarchical and complex designs, identification of the appropriate level for tests and full reporting of outcomes
☒	☐	Estimates of effect sizes (e.g. Cohen's *d*, Pearson's *r*), indicating how they were calculated

Our web collection on statistics for biologists contains articles on many of the points above.

Software and code

Policy information about availability of computer code

Data collection	Biaevaluation Biacore (see methods for detail)
Data analysis	GraphPhad Prizm 8.0.2, Flow Jo, pymol, UCSF Chimera 1.8, biaevaluation

For manuscripts utilizing custom algorithms or software that are central to the research but not yet described in published literature, software must be made available to editors/reviewers. We strongly encourage code deposition in a community repository (e.g. GitHub). See the Nature Research guidelines for submitting code & software for further information.

Data

Policy information about availability of data

All manuscripts must include a data availability statement. This statement should provide the following information, where applicable:

- Accession codes, unique identifiers, or web links for publicly available datasets
- A list of figures that have associated raw data
- A description of any restrictions on data availability

All data reported in this manuscript will be freely available.
The datasets generated during and/or analyzed during the current study are available from the corresponding author on reasonable request.
All data generated or analyzed during this study are included in this published article (and its supplementary information files).
The mass spectrometry proteomics data for lysine acetylation of TET2 post translational modifications have been deposited to the ProteomeXchange consortium (http://www.proteomexchange.org/submission/index.html via the PRIDE partner repository with the dataset identifier PXD020550.

Field-specific reporting

Please select the one below that is the best fit for your research. If you are not sure, read the appropriate sections before making your selection.

☒ Life sciences ☐ Behavioural & social sciences ☐ Ecological, evolutionary & environmental sciences

For a reference copy of the document with all sections, see nature.com/documents/nr-reporting-summary-flat.pdf

Life sciences study design

All studies must disclose on these points even when the disclosure is negative.

Sample size	All animal sample size was calculated by power calculation with keeping a difference of at least 25%
Data exclusions	None
Replication	Each experiments were done in triplicate at least two independent time
Randomization	Each set of animal experiment were randomized where ever required. This is stated in methods section.
Blinding	The blinding was not relevant to our study. Since all survival analysis were performed on different genotype of mice that were confirmed prior to treatment.

Reporting for specific materials, systems and methods

We require information from authors about some types of materials, experimental systems and methods used in many studies. Here, indicate whether each material, system or method listed is relevant to your study. If you are not sure if a list item applies to your research, read the appropriate section before selecting a response.

Materials & experimental systems

n/a	Involved in the study	
☐	☒	Antibodies
☐	☒	Eukaryotic cell lines
☒	☐	Palaeontology
☐	☒	Animals and other organisms
☒	☐	Human research participants
☒	☐	Clinical data

Methods

n/a	Involved in the study	
☒	☐	ChIP-seq
☐	☒	Flow cytometry
☒	☐	MRI-based neuroimaging

Antibodies

Antibodies used

Details are in the materials and methods and figure legends wherever necessary. Here are the details of that antibodies used in this study:
HRP-conjugated anti-rabbit secondary antibody (Santa Cruz, Cat# sc-2004, 1:10,000)
Goat anti Mouse IgG (H/L):HRP (Bio-Rad, Cat# STAR117P, 1:10,000)
anti-5hmC (Active motif, Cat# 39769, 1: 3,000);
anti Myc tag (Cell Signaling, Cat# 9402S, 1:3000);
anti-acetyl Lysine (Cell Signaling, Cat# 9441, 1:1000);
anti-5mC (Eurogentec, Cat# BI-MECY-0100, 1:2,500);
Anti-TET2 (Bethyl laboratories, Cat# 304-247A, 1:1000)
anti-Gr-1-PerCP, Clone RB6-8C5, BD Biosciences Cat# 552093, 1:200;
anti-MAC-1-PE, Clone M1/70, BD Biosciences Cat# 553311, 1:200).
anti-GST (Biolegend, Cat# 640802, 10 μg/ml)

Validation

All antibodies used are commercially available and validated.

Eukaryotic cell lines

Policy information about cell lines

Cell line source(s)

Details of cell lines are in the methods section. All cell lines were purchased recently and used within 10 passages in fresh culture. K562 cell lines were purchased from ATCC (Manassas, VA), while CMK, MEG-01, MOLM-13, HEL, OCI-AML5, and SIG-M5 were from DMSZ (Braunschweig, Germany) and grown according to the guidelines provided by them. Detailed information was provided in supplemental Table S1.

Authentication	Supplier, ATCC and DSMZ.
Mycoplasma contamination	Before freezing and after 5 splits, each cell lines used were tested routinely for mycoplasma contamination routinely using
Commonly misidentified lines (See ICLAC register)	none

Animals and other organisms

Policy information about studies involving animals; ARRIVE guidelines recommended for reporting animal research

Laboratory animals	C57Black6 mice, equal representation of male and female as far as practicable in each experiment.
Wild animals	None
Field-collected samples	none
Ethics oversight	Animal care and procedures was conducted in accordance with institutional guidelines approved by the Institutional Animal Care and Use Committee (IACUC) at Cleveland Clinic and also at university of Miami

Note that full information on the approval of the study protocol must also be provided in the manuscript.

Flow Cytometry

Plots

Confirm that:

☒ The axis labels state the marker and fluorochrome used (e.g. CD4-FITC).

☒ The axis scales are clearly visible. Include numbers along axes only for bottom left plot of group (a 'group' is an analysis of identical markers).

☒ All plots are contour plots with outliers or pseudocolor plots.

☒ A numerical value for number of cells or percentage (with statistics) is provided.

Methodology

Sample preparation	Methods section have the details
Instrument	BD FACSCantoII and LSRII flow cytometer.
Software	FlowJo7.6 software.
Cell population abundance	Details are in the methods section
Gating strategy	See methods for details and the figure.

☒ Tick this box to confirm that a figure exemplifying the gating strategy is provided in the Supplementary Information.
